# Supercritical CO_2_ Apple Pomace Extract: Chemical Composition, Antioxidant Activity, Cytocompatibility, Antibacterial and Antibiofilm Properties

**DOI:** 10.3390/foods15142435

**Published:** 2026-07-09

**Authors:** Gaia Muratore, Pierluigi A. Di Ciccio, Patrizia Morra, Eleonora Bianchi, Giuseppina Sandri, Giuseppe Mannino, Cinzia M. Bertea, Riccardo Destefano, Tiziana Civera

**Affiliations:** 1 Department of Veterinary Sciences, University of Turin, Largo Paolo Braccini 2, 10095 Grugliasco, TO, Italy; pierluigialdo.diciccio@unito.it (P.A.D.C.); patrizia.morra@unito.it (P.M.); tiziana.civera@unito.it (T.C.); 2 Department of Drug Sciences, University of Pavia, Via Taramelli 12, 27100 Pavia, PV, Italy; eleonora.bianchi01@unipv.it (E.B.); giuseppina.sandri@unipv.it (G.S.); 3 Department of Life Sciences and Systems Biology, University of Turin, Via Quarello 15/A, 10135 Torino, TO, Italy; giuseppe.mannino@unito.it (G.M.); cinzia.bertea@unito.it (C.M.B.); 4 Separeco S.r.l., Via Battitori 19, 10060 Macello, TO, Italy; destefano@separeco.com

**Keywords:** food by-products, apple pomace, bioactive compounds, supercritical fluid-assisted extraction, antibacterial activity, antibiofilm activity

## Abstract

Apple processing generates large amounts of pomace, a by-product rich in bioactive compounds. This study characterized an apple pomace extract (APE) obtained via supercritical CO_2_ and evaluated its antioxidant, cytotoxic, pro-inflammatory, antibacterial and antibiofilm properties. Total phenolic (TPC), proanthocyanidin (TPAC) and flavonoid (TFC) contents, together with DPPH, ABTS, FRAP assays, were determined in the starting material, exhausted residue and APE to assess extraction efficiency. Subsequently, the volatile and water-soluble fractions of the extract were analysed using GC-MS and HPLC-MS, respectively. Its safety was evaluated through a cytotoxicity assay on Caco-2/TC-7 cells and TNF-α secretion, while antibacterial and antibiofilm activities were assessed following a modified CLSI protocol and Innovotech guidelines, respectively. APE showed higher TPC, TPAC and TFC than both starting material and residue, together with strong antioxidant activity. Characterization revealed a predominance of pentacyclic triterpenes and glycosylated flavonols, while volatiles were dominated by alcohols and aldehydes. APE was cytocompatible up to 72 mg mL^−1^ and did not induce significant TNF-α release. It inhibited planktonic bacterial growth mainly at 90–180 mg mL^−1^, whereas biofilm eradication occurred at 45 mg mL^−1^. These findings support the use of apple pomace as a sustainable source of bioactive compounds for food applications.

## 1. Introduction

Apples (*Malus domestica*) are among the most widely cultivated and consumed fruits worldwide, with commercial production occurring in more than 90 countries [1,2]. In 2023, global apple production reached approximately 97 million tons, of which 17.5 million tons were produced in Europe [3]. Italy ranks as the second-largest producer in Europe and the eight-largest worldwide, with an estimated production of around 2.26 million tons [3]. Approximately 70–80% of total apple production is destined for fresh consumption, while the remaining fraction is processed industrially, primarily for juice production (accounting for about 70% of processed apples), cider, vinegar, purees, jams, dried fruit and pectin extraction [1].

The main by-product of the apple processing industry is apple pomace, a solid residue representing 25–30% of the fresh fruit weight and consisting predominantly of pulp, peels, seeds, and stems. The global annual production of apple pomace is estimated at approximately 4 million tons; however, despite its high availability, its recovery remains limited [4]. Currently, apple pomace is primarily disposed of in landfills, a practice that entails additional costs for the food industry and raises significant environmental concerns [4,5]. In recent years, increasing efforts have been directed toward the development of more sustainable strategies for the reuse and valorisation of this by-product [1,2]. Indeed, various applications have been reported in the literature, including its use as a fuel source, fertilizer and its incorporation into animal feed and food products [1,4,6]. Furthermore, growing interest has emerged in the recovery of bioactive compounds from agro-industrial by-products. In this context, apple pomace represents a valuable source of polyphenols and terpenoids, two classes of secondary plant metabolites. Regarding the polyphenolic fraction, the molecules most frequently identified include flavonoids, particularly flavonols, flavan-3-ols, anthocyanins and dihydrochalcones. Phenolic acids, mainly hydroxycinnamic acids, as well as tannins, have also been reported. In addition to polyphenols, terpenoids such as ursolic and oleanolic acids have been identified in apple pomace [7,8,9,10]. These molecules have been widely described in the literature for a variety of biological activities, particularly antioxidant and antibacterial, which are of considerable interest to the food industry, where oxidative processes and microbial contamination represent the main causes of food spoilage.

In recent years, extraction techniques for recovering bioactive compounds from agro-industrial by-products have undergone significant development [11]. Conventional extraction methods have been progressively complemented, and in some cases replaced, by innovative and more sustainable approaches developed to overcome several limitations associated with traditional processes [9,11,12]. These methods are often characterized by long extraction times, high solvent consumption, difficulties in obtaining high-purity extracts, potential degradation of thermolabile compounds, and limited selectivity toward the target molecules [12]. Emerging extraction technologies are capable of overcoming these limitations by reducing solvent consumption and processing time while improving extraction efficiency and selectivity [13]. Among these, supercritical fluid extraction (SFE) represents one of the most promising and widely applied technologies. SFE is based on the use of solvents subjected to temperature and pressure conditions above their critical point, at which the fluid exhibits properties intermediate between those of gases and liquids [14]. Among the solvents employed, CO_2_ is the most commonly used due to its high availability, low cost, non-toxicity, non-flammability and ease of removal from the final extract. This technology allows the production of extracts characterized by high-quality standards, particularly in terms of the purity and concentration of target compounds. Moreover, SFE is considered a sustainable and environmentally friendly extraction method, as CO_2_ can be easily recovered and recycled during the process. Owing to these characteristics, the technique has found wide application across several industrial sectors, including the food industry. Extracts obtained using supercritical CO_2_ are free from solvent residues, making them particularly suitable for food applications [13].

In light of these considerations, the present study aimed to evaluate the potential of apple pomace as a source of high-value bioactive compounds capable of improving food quality and safety. Specifically, the study intended to (i) characterize the phytochemical profile of an apple pomace extract obtained through supercritical CO_2_ extraction; (ii) assess its radical scavenging and reducing activities; (iii) evaluate its cytotoxicity and pro-inflammatory properties; and (iv) investigate its in vitro antibacterial and antibiofilm activities against both reference and wild-type strains relevant to the food sector, including spoilage bacteria and foodborne pathogens.

## 2. Materials and Methods

### 2.1. Plant Material and Extract Preparation

The extract employed in this work was obtained from pomace, a by-product of industrial apple processing consisting of seeds, pulp, petioles and peels in variable proportions. This residue, derived from different locally produced apple cultivar, was supplied in frozen form by Arc en Ciel Soc. Agr. Coop. (Cafasse, TO, Italy). It was dried in ovens at 60–62 °C, ground and subsequently stored at room temperature in the dark.

The resulting material was then subjected to supercritical CO_2_ extraction in the pilot-scale system of Separeco S.r.l. (Macello, TO, Italy). In particular, 1500 g of matrix were rehydrated with 23% (*w*/*w*) water (approximately 340 g), subjected to three 10 s cycles in a microwave at 800 W under controlled temperature conditions (<38 °C) and subsequently loaded in a 7 L extraction vessel equipped with two gravimetric separators. Extraction was performed at 60 °C and 250 bar for 1 h, with CO_2_ and ethanol (1 kg h^−1^) at a total flow rate of 20 kg h^−1^. Under these conditions, the extraction yield was 19.6%. The liquid extract, precipitated in the separators and characterized by a density of 0.9 g mL^−1^, was then collected in amber glass flasks.

The original plant matrix (apple pomace), together with the exhausted residue and the extract obtained by supercritical CO_2_ extraction, underwent preliminary spectrophotometric analyses to evaluate extraction yield and process efficiency in terms of bioactive compound content, as described in Section 2.2, Section 2.3, Section 2.4 and Section 2.5. All analyses were performed in technical triplicate.

### 2.2. Total Phenolic Content (TPC)

Total phenolic content (TPC) was determined using the Folin–Ciocalteu (FC) colorimetric method, as described by Magara et al. [15]. Briefly, the FC reagent (Sigma-Aldrich, Milan, Italy) was mixed separately with each sample (apple pomace, exhausted residue and extract) and a 7.5% (*w*/*v*) sodium bicarbonate solution (VWR International, Milan, Italy) in a 1:10:5 ratio (*v*/*v*/*v*). The final volume was adjusted to 1 mL with distilled water. The mixture was vortexed and incubated at 80 °C for 1 min, and the absorbance was measured at 765 nm using a UV-Vis spectrophotometer (Shimadzu, Milan, Italy) with 1 cm PMMA cuvettes. Gallic acid (≥99%, analytical grade) was used as the reference standard for constructing the calibration curve, as previously described by Mannino et al. [16]. TPC was expressed as mg gallic acid equivalents (GAE) per gram of dry weight (DW).

### 2.3. Total Proanthocyanidin Content (TPAC)

The total proanthocyanidin content (TPAC) was determined using the 4-(dimethylamino)cinnamaldehyde (DMAC) assay, according to the method described by Mannino et al. [16]. Samples (apple pomace, exhausted residue and extract) were diluted 1:10 (*v*/*v*) ratio in 75% (*v*/*v*) acetone containing 0.5% (*v*/*v*) acetic acid. Then, aliquots of 50 µL of each diluted sample were mixed with 150 µL of 0.1% (*w*/*v*) DMAC reagent prepared in 75% (*v*/*v*) ethanol acidified with 12.5% (*v*/*v*) HCl. After incubation for 20 min in the dark, absorbance was measured at 640 nm. The blank was prepared using the same reaction mixture and pure 75% (*v*/*v*) acetone acidified with 0.5% (*v*/*v*) acetic acid. Quantification was performed using an external calibration curve with pure A2-type proanthocyanidin (PAC-A2; Extrasynthese^®^, Genay, France) as the standard. Results were expressed as mg of PAC equivalents (PACE) per gram of dry weight (DW).

### 2.4. Total Flavonoid Content (TFC)

The total flavonoid content (TFC) was determined by an aluminum chloride colorimetric assay, according to the method described by Mannino et al. [17]. Aliquots of 40 µL of sample (apple pomace, exhausted residue and extract), appropriately diluted, were mixed with 6 µL of NaNO_2_ (5%, *w*/*v*) and incubated for 5 min. Subsequently, 6 µL of AlCl_3_ (10%, *w*/*v*) were added. After 6 min, 80 µL of NaOH (4%, *w*/*v*) and 68 µL of distilled water were dispensed. The samples were then mixed and incubated for a further 15 min. Absorbance was measured at 510 nm using a microplate reader (NB-12-0035; Neo Biotech, Nanterre, France). Flavonoid content was expressed as mg of rutin equivalents (RE) per gram of dry weight (DW). For this purpose, an external calibration curve of pure rutin (VWR International, Radnor, PA, USA), was used.

### 2.5. Antioxidant Activity

#### 2.5.1. DPPH Assay

Antioxidant activity was evaluated using the 2,2-diphenyl-1-picrylhydrazyl (DPPH^•^) assay [15]. A 0.1 mM methanolic DPPH solution (VWR International, Milan, Italy) was freshly prepared and kept in the dark until use. A 0.1 mL aliquot of appropriately diluted sample or Trolox (VWR International, Milan, Italy), used as the reference compound, was added to 3.9 mL of DPPH solution. The mixture was vortexed and incubated in the dark at room temperature for 30 min. The decrease in absorbance was measured at 517 nm using a UV-Vis spectrophotometer (Shimadzu, Milan, Italy). Radical scavenging activity was expressed as mmol of Trolox equivalents (TE) per gram of dry weight (DW), based on the calibration curve.

#### 2.5.2. ABTS Assay

Antioxidant activity was determined using the 2,2′-azinobis(3-ethylbenzothiazoline-6-sulfonic acid) (ABTS^•+^) assay [15]. The ABTS^•+^ radical was generated by reacting a 7 mM aqueous solution of ABTS (VWR International, Milan, Italy) with 2.45 mM potassium persulfate (VWR International, Milan, Italy) at a ratio of 10:1 (*v*/*v*), followed by incubation in the dark for 16 h at room temperature. Prior to analysis, the solution was diluted to obtain an absorbance of 0.70 ± 0.02 at 734 nm. A 0.1 mL aliquot of sample or Trolox (VWR International, Milan, Italy), used as the reference compound, was added to 3.9 mL of ABTS^•+^ solution and incubated for 6 min at room temperature. The reduction in absorbance was measured at 734 nm. Results were expressed as mmol of Trolox equivalents (TE) per gram of dry weight (DW).

#### 2.5.3. FRAP Assay

Reducing power was evaluated using the Ferric Reducing Antioxidant Power (FRAP) assay. The FRAP reagent was prepared fresh by mixing 300 mM acetate buffer (pH 3.6), 10 mM 2,4,6-tripyridyl-s-triazine (TPTZ) dissolved in 40 mM HCl, and 20 mM ferric chloride (FeCl_3_·6H_2_O) at a ratio of 10:1:1 (*v*/*v*/*v*), according to Magara et al. [15]. A 0.1 mL aliquot of sample or Trolox (VWR International, Milan, Italy), used as the reference standard, was added to 3.0 mL of FRAP reagent and incubated at 37 °C for 4 min. The increase in absorbance, corresponding to the formation of the Fe^2+^-TPTZ complex, was measured at 593 nm. Results were expressed as mmol of Trolox equivalents (TE) per gram of dry weight (DW).

### 2.6. Phenolic Compounds Identification and Quantification via High-Performance Liquid Chromatography

APE was also subjected to flavonoid profiling by high-performance liquid chromatography (HPLC) coupled with a diode array detector (DAD) and tandem mass spectrometry (MS/MS) with an electrospray ionization (ESI) source [17]. Analyses were performed using an Agilent 1260 Infinity HPLC system (Agilent Technologies, Santa Clara, CA, USA) coupled to an Agilent 6460 Ion Trap mass spectrometer (Bruker, Santa Clara, CA, USA). Chromatographic separation was performed on Zorbax Eclipse Plus C18 column (150 × 4.6 mm, 5 μm; Agilent Technologies) maintained at 30 °C. Elution was performed at a flow rate of 0.8 mL min^−1^ using the following gradient program: 0–5 min, 5% B; 5–20 min, 5–30% B; 20–30 min, 30–60% B; 30–35 min, 60–95% B; 35–40 min, 95% B, followed by re-equilibration to the initial conditions. The injection volume was 10 μL. DAD chromatograms were acquired between 200 and 600 nm, while MS/MS detection was performed in negative ionization mode (ESI-) under optimized source conditions, as previously described [18]. In order to ensure adequate resolution of the flavonoids present in the extracts, compounds were identified based on retention times, UV-Vis spectra, MS/MS fragmentation patterns, as well as by comparison with authentic standards and/or literature data. Analyses were performed in technical triplicate. Results were expressed as μg or mg per gram of dry weight (DW).

### 2.7. Profile of Volatile Compounds Using Gas Chromatography–Mass Spectrometry

The volatile compounds were analysed using gas chromatography coupled with mass spectrometry (GC-MS), after the sample was diluted 1:10 (*v*/*v*) in hexane. Analysis was conducted using a GC system equipped with a mass spectrometer and an electron impact (EI) ionization source operating at 70 eV, in accordance with established methods reported in the literature [19]. The separation of the compounds was carried out on a fused silica capillary column coated with a low-polarity stationary phase (30 m × 0.25 mm i.d., 0.25 μm film thickness), maintained under controlled thermal conditions. The sample was injected in splitless mode (1 μL injection volume; injector temperature 250 °C) and chromatographic separation was achieved using an optimized temperature program [19], with high-purity (helium, 99.999%) carrier gas maintained at a constant flow rate. Mass spectra were acquired in full-scan mode over the *m*/*z* range 35–500, while the transfer line and ion source temperatures were maintained at 280 °C and 230 °C, respectively. Volatile compounds were identified by comparing mass spectra with those present in commercial spectral libraries (NIST), and by comparing the retention indices calculated with literature data. Analyses were performed in technical triplicate. Results were expressed as μg or mg per gram of dry weight (DW). Relative quantification was performed via gas chromatography with flame ionization detection (GC-FID). The relative abundance of each compound was calculated from its peak area and expressed as a percentage (%) of the total chromatographic area, without the use of response factor corrections.

### 2.8. Cytotoxicity Assay and Pro-Inflammatory Response

Cytotoxicity was evaluated using the human colon adenocarcinoma Caco-2/TC-7 cell line (Merck, Milan, Italy), as previously described by Muratore et al. [20]. Cells were cultured in Dulbecco’s Modified Eagle Medium (DMEM) supplemented with 10% (*v*/*v*) fetal bovine serum (FBS; Euroclone, Milan, Italy), 200 IU mL^−1^ of penicillin and 0.2 mg mL^−1^ of streptomycin (Sigma-Aldrich, Milan, Italy). Once grown, Caco-2 cells were seeded in 96-well plates at a density of 3 × 10^4^ cells/well and incubated for 24 h at 37 °C in an atmosphere containing 5% CO_2_. After incubation, the culture medium was removed and cells were exposed for 24 h to 200 µL of freshly prepared extract solutions, obtained by dissolving APE at different concentrations in DMEM. Growth medium (GM) and 10% (*v*/*v*) Triton X-100 (Fluka, Milan, Italy) were used as positive and negative controls, respectively. After treatment, the medium was discarded and 100 µL of MTT solution was added to each well. Cells were then incubated at 37 °C for 3 h. The resulting formazan crystals were solubilized in dimethyl sulfoxide (DMSO; Carlo Erba, Milan, Italy), and absorbance was measured at 570 nm using a FLUOstar^®^ Omega Microplate Reader (BMG Labtech, Ortenberg, Germany). Results were obtained from five independent biological replicates and expressed as cell viability percentages relative to the control.

In addition, the pro-inflammatory response was evaluated by measuring TNF-α release using a commercial ELISA kit (Human TNF-α Conferma ELISA™; Merck Millipore, Burlington, MA, USA). Supernatants were collected after 3 h of treatment with the extract solutions, and cytokine levels were quantified at 450 nm. The assay was linear in the concentration range from 1.65 to 400 pg mL^−1^ (R^2^ > 0.995). Lipopolysaccharide (LPS, 2 µg mL^−1^ for 3 h) was used as positive control, while cells maintained in growth medium (GM) and 10% (*v*/*v*) Triton X-100 served as negative controls. Results were obtained from five independent biological replicates and expressed in pg mL^−1^.

### 2.9. In Vitro Assessment of the Antibacterial Activity

The antibacterial activity of APE was evaluated against both Gram-positive (*B. cereus* CIP 66.24T, *B. cereus* 117, *E. faecium* CIP 110055, *E. faecium* 1, *L. innocua* ATCC 33090, *L. innocua* 2, *L. monocytogenes* ATCC 13932, *L. monocytogenes* 3, *S. aureus* CIP 65.8T, *S. aureus* 2) and Gram-negative bacteria (*E. coli* CIP 105215, *E. coli* 1, *S. enterica* ser. Derby CIP 60.62T, *S. enterica* ser. Derby 2, *P. fluorescens* CIP 106483, *P. fluorescens* C2 74475). All strains were obtained from the Pasteur Institute (Paris, France) or obtained from the culture collection of the Department of Veterinary Sciences (University of Turin, Turin, Italy).

Prior to each experiment, 50 µL of the selected bacterial cultures were revived in 5 mL of fresh Mueller Hinton broth (MHB; Microbiol, Cagliari, Italy) and incubated for 24 h under optimal growth conditions (see Table 1). The optical density of the bacterial suspension was then measured using a spectrophotometer at λ = 640 nm, and the number of cells adjusted to 10^5^ CFU (Colony-Forming Unit) mL^−1^. These bacterial inocula were used to assess the antibacterial properties of the apple pomace extract. To this purpose, a broth microdilution test was performed according to the CLSI (Clinical and Laboratory Standards Institute) protocol [21], with some modifications made necessary to overcome interference in spectrophotometric readings caused by the pigmentation of the extract. In regard to this, microtubes were adopted instead of 96-well plates, as well as plate count agar on selective/differential medium. Briefly, 100 µL of bacterial inoculum was inoculated in microtubes already containing an equal volume of two-fold serially diluted APE ranging in concentrations from 22.5 to 180 mg mL^−1^. Three technical replicates were set up for each tested extract concentration. In addition, two controls were established: a positive, consisting of culture medium and bacterial suspension, and a negative, constituted only of fresh MHB. After 24 h of incubation at the optimal growth temperature, 100 µL from each microtube were plated on a selective/differential medium and incubated at the same conditions. The lowest APE concentration, expressed in mg mL^−1^, at which no visible bacterial growth occurred was defined as the minimum inhibitory concentration (MIC).

### 2.10. Minimum Biofilm Eradication Assay

The ability of APE to eradicate pre-formed biofilms was evaluated by determining the minimum biofilm eradication concentration (MBEC) as defined by the standardised Innovotech protocol for biofilm disruption studies [22]. For this purpose, only bacterial strains previously identified as biofilm producers were used in the assay [20].

Bacterial suspensions were adjusted to 10^5^ CFU mL^−1^, and 150 µL were inoculated into 96-well flat-bottom microtiter plates, subsequently covered with an uncoated 96-peg lid (MBEC Assay ^®^ Biofilm Inoculator with 96 Well Base; Innovotech Inc., Edmonton, AB, Canada). The plates were incubated at 37 °C for 24 h in a shaking water bath (SW22; Julabo ^®^ GmbH, Seelbach, Germany) set to 110 revolutions per minute (rpm) to allow biofilm formation. Following incubation, the pegs were rinsed in 200 µL of phosphate-buffered saline (PBS; Microbiol, Cagliari, Italy) to remove planktonic cells and transferred to challenge plates containing 200 µL per well of APE at different concentrations (45–360 mg mL^−1^, in triplicate). The plates were incubated for 24 h under appropriate growth conditions for each bacterial strain. In parallel, biofilm formation was confirmed prior to treatment by detaching cells from selected pegs (biofilm growth check, BGC) via sonication (30 min at 99 Hz; Ultrasonic Cleaner CP102; CEIA, Arezzo, Italy). Aliquots of 100 µL of the resulting suspensions were plated on selective/differential agar and incubated for 24–48 h at the same conditions. The presence of bacterial growth confirmed correct biofilm formation. After exposure, the pegs were rinsed for 1 min as described above and transferred to recovery plates containing 200 µL per well of fresh Brain Heart Infusion (BHI) broth (VWR International, Radnor, PA, USA). The biofilms were detached by sonication for 30 min at 99 Hz (Ultrasonic Cleaner CP102; CEIA, Arezzo, Italy), and the recovery plates were incubated for 24 h under strain-specific growth conditions. Subsequently, 100 µL of each technical replicate were plated on selective/differential agar and incubated for 24–48 h. The MBEC was defined as the lowest concentration of APE (mg mL^−1^) resulting in no visible growth on agar plates.

In parallel, the minimum biocidal concentration (MBC) was determined by transferring 20 µL from each well of the challenge plate into a new sterile 96-well microtiter plate prefilled with 180 µL of fresh BHI broth. The plates were incubated for 24 h under the optimal growth conditions for each bacterial strain. After incubation, 100 µL of each technical replicate were plated on selective/differential agar and incubated for 24–48 h. The MBC was defined as the lowest APE concentration (mg mL^−1^) capable of killing 99.9% of the dispersed bacterial cells released from the biofilm.

### 2.11. Statistical Analysis

Statistical differences were evaluated using one-way analysis of variance (ANOVA), followed by Tukey’s or Scheffé’s post hoc test. A *p*-value < 0.05 was considered statistically significant. All statistical analyses were performed using SPSS v. 28 software (IBM Corp, Armonk, NY, USA).

## 3. Results

### 3.1. CO_2_ Extraction, Yield and Bioactivity

**Figure 1:**
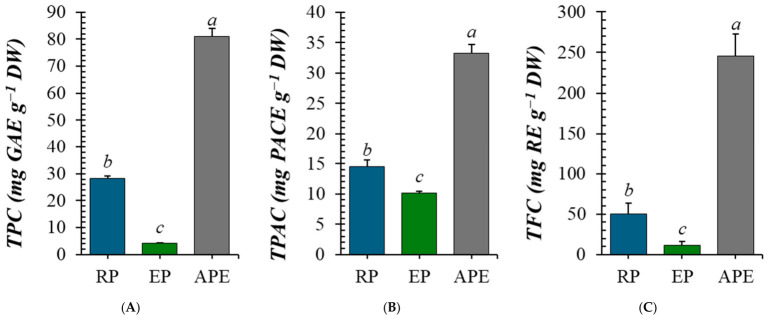
Content of bioactive compounds of raw apple pomace (RP), exhausted pomace (EP) and supercritical CO_2_ extract (APE). (**A**) Total phenolic content (TPC) expressed as mg GAE g^−1^ DW. (**B**) Total proanthocyanidins content (TPAC) expressed as mg PACE g^−1^ DW. (**C**) Total flavonoid content (TFC) expressed as mg RE g^−1^ DW. Results are expressed as mean ± standard deviation. Different lowercase letters indicate statistically significant differences, as determined by one-way ANOVA followed by Tukey’s post hoc test.

The total content of phenolic compounds (mg GAE g^−1^ DW), proanthocyanidins (mg PACE g^−1^ DW), and flavonoids (mg RE g^−1^ DW) in raw apple pomace (RP), exhausted pomace (EP), and supercritical CO_2_ extract (APE) are reported in Figure 1A–C.

RP showed a TPC of 28.21 mg GAE g^−1^ DW, along with 14.55 mg PACE g^−1^ DW and 50.26 mg RE g^−1^ DW. After extraction, EP exhibited a marked decrease in all measured bioactive compounds, with values of 4.09 mg GAE g^−1^ DW, 10.19 mg PACE g^−1^ DW and 11.10 mg RE g^−1^ DW, respectively. In contrast, APE was characterized by a significant increase in all parameters, reaching 80.97 mg GAE g^−1^ DW, 33.20 mg PACE g^−1^ DW and 245.95 mg RE g^−1^ DW. Notably, TPC in APE was approximately threefold higher than in the starting material (RP), while flavonoids increased by about fivefold.

With regard to antioxidant activity (Table 2), a similar trend was found in all tests. In the FRAP assay, APE exhibited the highest value (306.15 mmol TE g^−1^ DW), followed by RP (201.14 mmol TE g^−1^ DW), whereas a substantial reduction in activity was observed for EP (16.49 mmol TE g^−1^ DW). This pattern was further confirmed by the DPPH and ABTS radical scavenging assays. In the DPPH assay, APE exhibited the highest antioxidant activity (123.25 mmol TE g^−1^ DW). RP showed intermediate activity (43.50 mmol TE g^−1^ DW), whereas the EP displayed the lowest antioxidant capacity (12.27 mmol TE g^−1^ DW). A similar trend was observed in the ABTS assay. APE exhibited the highest antioxidant activity corresponding to 254.33 mmol TE g^−1^ DW. In contrast, RP and EP exhibited markedly lower antioxidant activity (19.54 and 14.32 mmol TE g^−1^ DW, respectively).

### 3.2. Hydrosoluble Bioactive Compounds in APE

**Figure 2:**
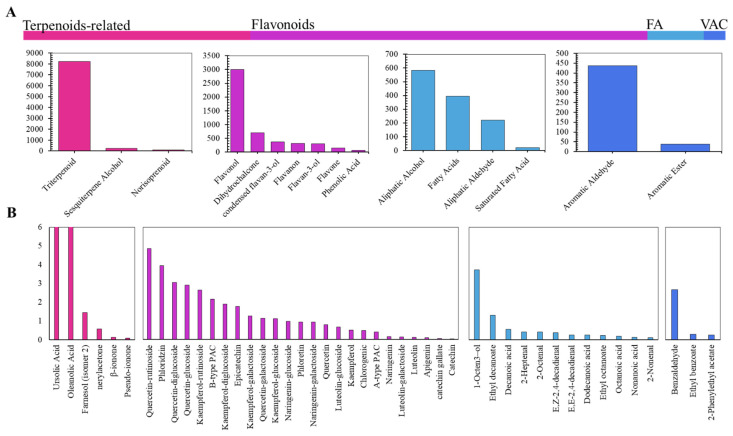
Graphical representation of the phytochemical profile of apple pomace extract (APE). (**A**) Distribution of identified secondary metabolites, grouped according to the major polyphenol classes. (**B**) Detailed classification, in which individual bars correspond to specific compounds and are colour-coded to indicate their assignment to the different subclasses reported in panel (**A**).

HPLC analysis allowed the identification and quantification of 26 compounds, with a total content of 12.57 ± 1.24 mg g^−1^ (Figure 2). The phytochemical profile was characterized by a marked prevalence of pentacyclic triterpenes and, among flavonoids, by a predominance of glycosylated forms over aglycones.

Pentacyclic triterpenes were the predominant fraction, with a total content of 7.77 mg g^−1^, corresponding to approximately 61% of the total quantified compounds. Ursolic acid was the most abundant compound (4.62 ± 0.61 mg g^−1^), followed by oleanolic acid (3.15 ± 0.16 mg g^−1^). Flavonols represented the second most abundant class (2.93 mg g^−1^; about 23%), mainly consisting of quercetin derivatives (1852.11 µg g^−1^) and kaempferol derivatives (1082.05 µg g^−1^). Among quercetin derivatives, quercetin-rutinoside was the most abundant compound (730.00 ± 130.02 µg g^−1^), followed by quercetin-diglucoside (433.50 ± 15.53 µg g^−1^) and quercetin-glucoside (411.74 ± 26.38 µg g^−1^). Kaempferol derivatives were mainly represented by kaempferol-rutinoside (375.04 ± 39.47 µg g^−1^). Flavan-3-ols and proanthocyanidins accounted for 624.62 µg g^−1^ (approximately 5%), with a prevalence of B-type proanthocyanidins (301.34 ± 22.02 µg g^−1^) and epicatechin (250.47 ± 34.50 µg g^−1^). Dihydrochalcones (phloridzin and phloretin) accounted for 718.56 µg g^−1^ (about 6%), mainly represented by phloridzin (583.46 ± 32.15 µg g^−1^). Flavanones (naringenin and derivatives) and flavones (luteolin, apigenin, and derivatives) were present in lower amounts: 300.71 µg g^−1^ (about 3%) and 127.58 µg g^−1^ (approximately 1%), respectively. Phenolic acids, represented by chlorogenic acid, showed the lowest content of 72.50 ± 2.78 µg g^−1^ (about 0.6%). Regarding glycosylation degree, glycosylated flavonoids (mono-, di-, and rutinosides) represented the predominant fraction (80–85%), while aglycones were present at lower levels (15–20%). In particular, rutinosides (quercetin- and kaempferol-rutinoside) were the most abundant glycosylated flavonols, followed by mono-glucosides, mono-galactosides, and diglucosides.

### 3.3. Volatile Compounds Characterization in APE

GC analysis allowed the identification and quantification of 19 volatile compounds, with a total content of 1.91 ± 0.19 mg g^−1^ (Table 3). The most abundant compounds were 1-octen-3-ol (506.89 ± 53.314 µg g^−1^), benzaldehyde (389.50 ± 28.06 µg g^−1^), farnesol (isomer 2) (205.48 ± 36.27 µg g^−1^), and ethyl decanoate (187.14 ± 27.25 µg g^−1^), collectively accounting for approximately 68% of the total identified volatiles.

Among the different chemical classes, alcohols (mainly 1-octen-3-ol and farnesol) constitute the predominant fraction (approximately 37%), followed by aldehydes (about 32%), mainly including benzaldehyde and unsaturated C6–C10 derivatives (2-nonenal, 2-octenal, 2-heptenal, and E, E-/E, Z-2,4-decadienal). Esters (ethyl decanoate, ethyl benzoate, 2-phenylethyl acetate, and ethyl octanoate) contributed about 16%, whereas fatty acids (octanoic, nonanoic, decanoic, and dodecanoic) accounted for approximately 9% of the total. The ketone nerylacetone and the norisoprenoids (pseudo-ionone and β-ionone) contributed to a lesser extent, accounting for about 4% and 1.5%, respectively.

### 3.4. Cytotoxicity Assay and Pro-Inflammatory Immune Response of APE

**Figure 3:**
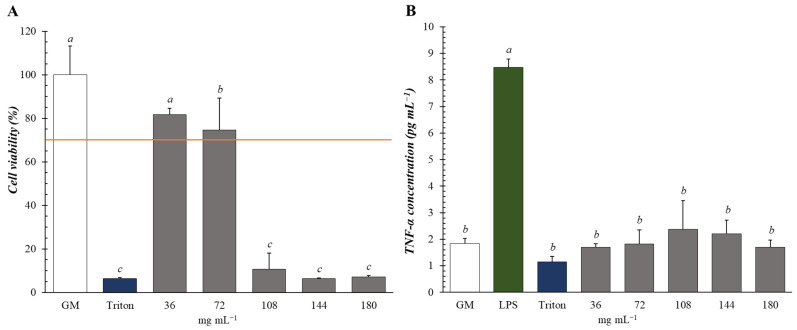
Cytotoxicity and pro-inflammatory response induced by different concentrations of supercritical CO_2_ extract (APE). (**A**) Viability of Caco-2/TC-7 cells expressed as percentage (%). The orange line indicates the 70% viability threshold. (**B**) TNF-α secretion by Caco-2/TC-7 cells expressed in pg mL^−1^. Data are reported as mean ± standard deviation. Different lowercase letters indicate statistically significant differences as measured by one-way ANOVA followed by Scheffé’s post hoc test.

The effect of increasing concentrations of APE on Caco-2/TC-7 cell viability after 3 h of exposure, assessed by the MTT assay, is shown in Figure 3. A significant reduction in cell viability (*p* < 0.05) was observed at 72 mg mL^−1^ compared to the positive control (growth medium, GM). Nevertheless, the extract was cytocompatible with the selected cell line at the lowest concentrations tested (36 and 72 mg mL^−1^), at which cell viability remained above 70% (as recommended by ISO guideline 10993-Biological evaluation). In contrast, APE exhibited cytotoxic effects at concentrations starting from 108 mg mL^−1^.

Additionally, no inflammatory response was observed following exposure to both the cytocompatible concentrations of APE (36 and 72 mg mL^−1^). Indeed, all TNF-α levels were comparable to those of the non-inflamed control and significantly lower (*p* < 0.05) than those of LPS-treated samples.

### 3.5. Determination of Antibacterial Activity of APE

Table 4 summarizes the results of the antibacterial activity screening of the apple pomace extract, expressed as minimum inhibitory concentration (MIC) values. Among the tested bacterial species, the reference strain of *P. fluorescens* and the wild-type strain of *E. coli* were the most susceptible, showing growth inhibition at an extract concentration of 45 mg mL^−1^. All other selected strains were inhibited only at higher extract concentrations, specifically at 90 or 180 mg mL^−1^. By contrast, the wild-type strain of *B. cereus* displayed no detectable inhibition at any of the tested concentrations.

### 3.6. Minimum Biofilm Eradication Assay

Exposure to apple pomace extract inhibited the normal development of biofilms at a concentration of 45 mg mL^−1^ for all tested biofilm-forming strains, with the only exception of the reference strain of *S. enterica* ser. Derby (CIP 60.62T), whose aggregated form did not respond to any of the concentrations assayed (Table 4).

Regarding MBC, cells of all the biofilm-forming strains detached from the biofilm responded to an extract concentration of 45 mg mL^−1^, with the exception of those of the reference strain of *S. enterica* ser. Derby (CIP 60.62T), which were resistant to all concentrations tested.

## 4. Discussion

Apples are among the most widely cultivated fruits worldwide. The majority are consumed fresh, while a significant proportion is destined for industrial processing. The main by-product is apple pomace, consisting of pulp, peels, seeds and stems, whose management is still largely based on landfill disposal, resulting in additional costs for companies and environmental concerns. However, in recent years, increasing attention has been directed toward the potential of this by-product as a source of bioactive compounds, particularly polyphenols and terpenoids, which are well known for their antioxidant and antimicrobial properties. These molecules therefore represent a promising natural alternative to synthetic preservatives in the food sector, as they could be used to inhibit and control the growth of spoilage and pathogenic bacteria, thereby extending food shelf life and improving food safety. Through the use of advanced extraction technologies, it is now possible to obtain extracts rich in these compounds, characterized by high purity and suitability for food applications. However, the chemical composition of apple pomace may be influenced by several factors, including apple cultivar, environmental and climatic conditions during cultivation, and the extraction technology employed. Consequently, an in-depth characterization of each extract is essential.

Accordingly, the phytochemical profile of APE was first investigated to provide a basis for interpreting its biological properties, including antioxidant activity, cytotoxicity, pro-inflammatory response, antibacterial and antibiofilm potential. Preliminary spectrophotometric analyses indicated that the supercritical CO_2_ extraction process effectively concentrated phenolic compounds from apple pomace, as reflected by their depletion in the residual solid matrix (Figure 1). However, the less pronounced reduction in type A proanthocyanidins in the spent material suggests a lower extractability of this class of compounds compared with the other phenols analysed. Chromatographic analyses confirmed that apple residues represent a high added-value matrix. In particular, supercritical CO_2_ technology allowed the selective recovery of lipophilic bioactive constituents, especially triterpenes, while preserving a significant proportion of phenolic compounds together with an aromatically active volatile fraction. These findings support the potential of this extraction approach within circular economy strategies applied to the agri-food sector, where the recovery of molecules with high nutraceutical value from industrial by-products is increasingly recognized as a priority [9,23]. In particular, this study demonstrates that supercritical CO_2_ extraction applied to apple waste residues yields an extract characterized by a high content of bioactive compounds, with a marked predominance of pentacyclic triterpenes and a significant presence of glycosylated flavonoids, associated with significant antioxidant activity. The total phenolic content (80.97 mg GAE g^−1^ DW) obtained in this study is higher than the values commonly reported for apple by-products extracted using conventional hydroalcoholic solvent systems, which typically range between 10 and 25 mg GAE g^−1^ [2,24,25]. However, some higher values have also been reported in the literature. For instance, da Silva et al. [26] observed TPC levels of up to 148 and 221 mg GAE g^−1^ DW in apple by-products extracted via conventional maceration and Soxhlet extraction using hydroalcoholic solvents. These findings suggest that the extraction yield may be influenced by process parameters, including solvent type and extraction conditions. In the present work, the relatively high TPC may be associated with the use of a supercritical CO_2_ technology, which, although selective towards compounds of moderate polarity, may enable the recover a significant proportion of antioxidant molecules.

The same trend observed for TPC was also found for TFC and TPAC, corresponding to flavan-3-ol polymers and oligomers detectable by the DMAC assay. In particular, compared with the exhausted material after supercritical fluid extraction, TFC showed an increase of approximately fivefold, whereas PACs increased by about twofold (Figure 1B,C). Although the magnitude of the increase differed between the two classes, the values detected in the spent residue confirm that the extraction process was effective in recovering significant amounts of both polyphenolic fractions. Among these, PACs are particularly relevant as they are widely associated with several biological activities of interest, including antimicrobial activity [27]. This property has been attributed to their ability to interact with microbial cell membranes, alter their permeability, form complexes with essential proteins and enzymes, and interfere with adhesion and biofilm formation [28]. Therefore, the enrichment of the extract in PACs, although quantitatively less pronounced than that observed for total flavonoids, may represent a relevant contributor to the functional enhancement of the obtained extract.

Consistently with its phytochemical composition, APE showed antioxidant activity. The higher ABTS values compared with those obtained by DPPH and FRAP assays suggest the presence of antioxidant compounds acting through different redox mechanisms. It is known that the ABTS assay responds to a broader spectrum of antioxidants than DPPH, while FRAP mainly measures reducing power [29]. The trend observed is consistent with that reported for apple peel and pomace extracts, in which scavenger activity is strongly correlated with the presence of flavonols and proanthocyanidins [30]. However, in the specific case of supercritical CO_2_ extraction, the high concentration of ursolic acid and oleanolic acid represents a distinctive feature compared with traditional hydroalcoholic extracts, which tend to be enriched in more polar fractions. Literature evidence indicates that pentacyclic triterpenes are particularly abundant in the cuticle of apple peel and are more efficiently extractable with low-polarity solvents or supercritical technologies [31,32]. The predominance of glycosylated forms of quercetin and kaempferol (approximately 80–85% of total flavonoids) is consistent with the typical phytochemical profile of apple tissues [33,34]. Although aglycones generally exhibit higher specific antioxidant activity in vitro, the prevalent presence of glycosides reflects the natural state of the plant matrix and may positively influence both stability and bioavailability [35]. Also of interest is the concentration of phloridzin, a dihydrochalcone characteristic of the genus *Malus*, which has been widely reported in apple by-products and associated with potential modulatory effects on carbohydrate metabolism [36,37,38].

From an aromatic perspective, the volatile profile is dominated by 1-octen-3-ol, benzaldehyde, farnesol, and ethyl esters. The significant presence of 1-octen-3-ol and unsaturated C6–C10 aldehydes indicates activation of the lipoxygenase pathway, already documented in apple tissues following cell disruption or enzymatic oxidation processes [39,40,41]. Benzaldehyde, commonly associated with almond-like notes, has previously been identified in apple-derived products and may originate from the degradation of phenylpropanoid precursors [40,42,43]. The detection of norisoprenoids such as β-ionone, albeit in modest concentrations, is noteworthy as these compounds, derived from carotenoids degradation [44], possess very low olfactory thresholds and therefore contribute significantly to the overall aromatic bouquet.

To provide a comprehensive assessment of APE, its safety was also investigated through in vitro evaluation of cytotoxicity in an intestinal cell line, together with its potential pro-inflammatory response. Cytotoxicity toward Caco-2/TC-7 cells at concentrations higher than 108 mg mL^−1^ could be associated with the presence of pentacyclic triterpenes, in particular ursolic and oleanolic acid. Indeed, these compounds have been reported to exert antitumor-related effects in vivo, including apoptosis induction, cell cycle arrest, autophagy, inhibition of invasion, metastasis, and angiogenesis [45]. The mixture of these two molecules has been reported to exhibit significant cytotoxicity against Caco-2 cells at concentrations starting from 16 μg L^−1^ for both compounds [46]. Similarly, quercetin and kaempferol have been described as cytotoxic toward Caco-2 cells at concentrations of 7.6 μg L^−1^ and 5 μg L^−1^, respectively [47]. However, it should be noted that the Caco-2 model may exhibit higher sensitivity compared with in vivo or ex vivo substrate, as the cells lack the protective mucus layer that normally covers the intestinal epithelium. Moreover, presystemic metabolism is not present in this in vitro system, leading to direct exposure of the cells to the tested compounds. Overall, these observations support the preventive effect of APE in protecting the intestine against tumorigenesis. Despite the cytotoxic effects observed at higher concentrations, at biocompatible levels the extracts did not induce an inflammatory response, which may be conceivably attributable to the synergic effect of pentacyclic triterpenes and flavonoids [45,46,47].

Finally, the evaluation of APE antibacterial activity against planktonic bacteria revealed an overall inhibitory effect against almost all tested strains, with more pronounced activity observed against the reference strain of *P. fluorescens* and the wild-type strain of *E. coli*. The only exception was the wild-type strain of *B. cereus*, whose growth was not inhibited at the extract concentrations tested. The high susceptibility of *P. fluorescens* and *E. coli* strains, for which antibacterial activity was already evident at a concentration of 45 mg mL^−1^, is in agreement with the findings reported by Raphaelli et al. [48]. Specifically, these authors described a marked antibacterial activity of an apple pomace extract obtained from the Gala cultivar through organic solvent extraction (acetone: ethanol) against *E. coli*, *S. typhimurium* and *S. aureus*. Similar observations were reported by Zambrano et al. [49], who highlighted a greater efficacy of different apple pomace extracts against species belonging to the *Pseudomonas* genus, including *P. putida* and *P. aeruginosa*, as well as strains of the *Bacillus* genus. These results do not fully reflect the behaviour commonly reported in the literature. Indeed, Gram-negative bacteria are generally described as more resistant to plant-derived extracts due to the greater structural complexity of their cell wall and the presence of an outer membrane [50]. The pronounced susceptibility observed in the present study may be attributed to the phenolic composition of the apple pomace extract, which likely contains bioactive compounds capable of interacting with the membrane structures of Gram-negative bacteria. For instance, Fariñas-Mera et al. [2] suggested that the enhanced activity of the extract could be attributed to its relatively high flavonoid content, particularly phloridzin and phloretin. These compounds have been reported to exert significant antibacterial effect against microorganisms such as *E. coli* [51]. Finally, the different behaviour exhibited by the wild-type *B. cereus* strain could be related to its origin. Indeed, environmental isolates are exposed to variable and stressful conditions, which may promote the development of specific adaptive mechanisms [52]. In contrast, laboratory reference strains are typically maintained under stable and controlled growth conditions. Therefore, niche-specific adaptation may have influenced the different response observed in the present study.

Although APE exhibited antibacterial activity against the planktonic forms of the tested strains, microbial contamination in the food sector is more commonly associated with bacterial biofilms. Indeed, these microbial communities, composed of spoilage and/or pathogenic bacteria embedded in a self-produced extracellular polymeric substance matrix, exhibit increased tolerance to antimicrobial agents, thereby promoting bacterial persistence in food processing environments [53,54,55]. Consequently, biofilms represent a major concern for the food industry, as they act as persistent reservoirs of contamination and may poses a potential threat to public health [55]. In light of these considerations, the evaluation of the antibiofilm activity of the apple pomace extract represents a crucial step in complementing the data obtained on planktonic forms and in further elucidating its potential application in the food sector. The results of the antibiofilm activity assessment, conducted according to the Innovotech protocol, indicated that APE was able to eradicate the biofilm of all tested strains, with the exception of *S. enterica* ser. Derby. In this case, the aggregated form of the strain was not eradicated at any of the extract concentrations evaluated. A particularly noteworthy finding emerged from the comparison between MBEC and minimum inhibitory concentration (MIC) values: for nearly all strains, the MBEC was lower than the MIC. This finding appears counterintuitive, considering that biofilm formation represents a growth strategy aimed at enhancing bacterial persistence under unfavourable environmental conditions. However, the phytochemical composition of APE may provide some insights into the observed antibiofilm activity. Different studies have indeed reported that bioactive compounds present in fruit and vegetable by-products, as well as in their derived extracts, may interfere with different stages of biofilm development. Among these, in the case of apple, particular attention has been given to ursolic and oleanolic acids, which have been described as modulators of the expression of genes involved in biofilm formation [56]. In addition, several phenolic compounds naturally occurring in apples, including quercetin, proanthocyanidins, rutin, phloretin and chlorogenic acid, have been associated with antibiofilm activity. The proposed mechanisms primarily involve interference with quorum sensing systems and the inhibition of the initial adhesion of bacterial cells to surfaces [57,58,59]. Despite this evidence, studies specifically investigating the antibiofilm activity of extracts obtained from apple processing by-products remained limited, making direct comparisons with the findings of the present study difficult. Among the few available studies, Fariñas-Mera et al. [2] investigated the antibiofilm activity of an extract obtained using conventional extraction method from apple processing residues focusing primarily on its ability to inhibit biofilm formation. Specifically, the authors reported complete inhibition of biofilm formation by the three bacterial strains investigated, namely *A. caviae*, *L. monocytogenes* and *P. syringae*, at concentrations corresponding to the MIC. In contrast, the present study evaluated the ability of APE to eradicate mature biofilms, as these represent a continuous source of dispersed cells, which play a crucial role in the colonization of new environments [60,61]. Accordingly, the susceptibility of biofilm-dispersed cells to APE was also investigated in order to explore its potential application in controlling the spread of contaminating microorganisms. The results demonstrated a marked bactericidal effect of the extract against dispersed cells of all the tested strains at a concentration of 45 mg mL^−1^. An exception was observed for *S. enterica*, whose dispersed cells did not show sensitivity at any of the tested concentrations. Overall, these findings suggest that dispersed cells retain a susceptibility profile more similar to that of the corresponding sessile forms than to that of their planktonic counterparts.

Therefore, given its demonstrated antioxidant, antibacterial and antibiofilm properties, the extract obtained by supercritical CO_2_ extraction shows considerable potential for future applications in the food sector, where it could contribute to the control of oxidative processes, the extension of product shelf life and the enhancement of microbiological safety.

However, one of the major challenges associated with the industrial use of plant-derived extracts is the standardization of their phytochemical composition. The profile and concentration of bioactive compounds may vary depending on the cultivar, the fruit ripening stage, the climatic growing conditions and the storage practices adopted for the raw material [33]. Consequently, fluctuations in the quantitative and qualitative content of bioactive compounds may result in differences in both efficacy and safety [62,63].

Furthermore, the effects observed in vitro at specific concentrations may not be directly reproduced in foods [63]. The structural complexity of food matrices may influence the distribution of bioactive compounds, thereby affecting their bioavailability and, ultimately, their efficacy [62,64]. Another critical factor is their stability during food processing and storage. Indeed, structural modifications may occur as a result of exposure to environmental conditions, such as pH, temperature, light, oxygen, metal ions, and enzymatic activity, which may compromise their biological activity [62,65]. Beyond degradation processes, bioactive compounds may also interact with food matrix constituents, including proteins, lipids and carbohydrates [62,63]. As a consequence, their biologically available fraction may be reduced, limiting their ability to act on target microorganisms [66]. In parallel, food matrix components may protect microbial cells by restricting the contact between the bioactive compounds and their cellular structures [66]. Finally, the incorporation of plant-derived extracts rich in bioactive compounds into food products may alter their sensory attributes such as flavour, aroma, colour and texture [67,68], which could negatively affect the overall consumer acceptance [69,70].

To address these limitations, several strategies have been developed in recent years. Among these, encapsulation represents one of the most widely investigated and suitable approaches for the protection and delivery of bioactive compounds extracted from fruits, vegetables and their by-products [71]. This technology involves entrapping the active compound within coating materials, which protect it from processing conditions and environmental factors responsible for its degradation and inactivation [72,73]. A variety of encapsulation systems are currently available [74]. The selection of the most appropriate strategy is crucial and should consider both the physicochemical properties of the target compound and those of the intended food matrix [75,76].

Although these technologies represent a promising approach for the application of plant extracts in food products, their associated costs may constitute a significant limitation, particularly for small and medium-sized enterprises [76]. These economic constraints arise across multiple stages of the valorisation chain of agro-industrial by-products. They include pre-treatment operations such as washing, cutting and drying, which are often required to improve the efficiency of bioactive compound recovery [77,78], as well as the extraction processes. Indeed, conventional extraction techniques are generally characterized by high operating costs [77], but even more advanced technologies such as supercritical fluid extraction require substantial investment due to the complexity of the equipment involved [79]. Therefore, comprehensive techno-economic analyses are essential to assess the overall feasibility of bioactive compound recovery from agro-industrial by-products. If these evaluations demonstrate a tangible application potential, the resulting extracts should undergo a regulatory pathway aimed at their authorization as food additives, similarly to what has been established for rosemary extract (E392) [80]. To this end, risk assessment conducted by the relevant regulatory authorities is necessary, considering the chemical composition of the extract, in order to ensure its safe use in food applications.

## 5. Conclusions

Overall, this study indicates that supercritical CO_2_ extraction enables the recovery of an extract enriched in bioactive compounds. Specifically, the total phenolic content, as well as the levels of proanthocyanidins and flavonoids, was higher in the apple pomace extract (APE) than in the raw material (RP), while being significantly reduced in the exhausted residue (EP). Antioxidant assays (FRAP, ABTS and DPPH) further confirmed the effectiveness of the extraction process, highlighting that the extract exhibited greater antioxidant activity than both the starting matrix and the residual by-product. Polyphenolic profiling and quantification provided deeper insight into the chemical composition of the extract, which was found to be rich in pentacyclic triterpenes and flavonoids, mainly in glycosylated forms. Additionally, volatile compounds were detected, primarily belonging to the classes of alcohols and aldehydes. Cytotoxicity assessment using the MTT assay identified two cytocompatible concentrations (36 and 72 mg mL^−1^), which were subsequently tested for their potential to induce an inflammatory response. TNF-α levels showed no increase, suggesting that, under the tested conditions, the extract does not trigger inflammation. Moreover, the extract demonstrated antibacterial activity against both Gram-positive and Gram-negative bacteria, and exhibited antibiofilm potential in most cases, even at sub-MIC concentrations. In conclusion, the findings obtained highlight the potential added value of apple processing by-products. In line with the principles of the circular economy, it is possible to recover extracts rich in bioactive compounds from these residues that exhibit notable antioxidant and antibacterial activities. These extracts therefore represent a promising natural alternative to synthetic food additives, with potential applications in the food industry aimed at extending shelf life by limiting the growth of spoilage bacteria and controlling foodborne pathogens, thereby improving food safety. Furthermore, their incorporation into food formulations may enhance the overall nutritional profile of food products.

## Figures and Tables

**Table 1:** Bacterial strains used to evaluate the antibacterial and antibiofilm activity of apple pomace extract, information on their isolation sources, the conditions required for their optimal growth and the selective/differential media used for culturing.

Bacterial Strains	Isolation Source	Growth Conditions	Selective/ Differential Medium
Gram-positive			
*B. cereus* CIP 66.24T	Unknown source	24 h at 30 °C	Bacillus cereus Selective Agar Base Oxoid, CM0617
*B. cereus* 117	Cheese		
*E. faecium* CIP 110055	Faeces, rectal swab	24 h at 37 °C	Kanamycin Aesculin Azide Agar Base Oxoid, CM0591
*E. faecium* 1	Cheese		
*L. innocua* ATCC 33090	Bovine encephalon	48 h at 37 °C	Chromogenic Listeria Agar (ISO) Base Oxoid, CM1084
*L. innocua* 2	Salmon		
*L. monocytogenes* ATCC 13932	Spinal fluid	48 h at 37 °C	Chromogenic Listeria Agar (ISO) Base Oxoid, CM1084
*L. monocytogenes* 3	Meat		
*S. aureus* CIP 65.8T	Pleural fluid	24 h at 37 °C	Baird-Parker Agar (ISO) Base Oxoid, CM1127
*S. aureus* 2	Chicken		
Gram-negative			
*E. coli* CIP 105215	Environment	24 h at 41 °C	Tryptone Bile X-Glucuronide (TBX) Medium Oxoid, CM0945
*E. coli* 1	Carcass		
*P. fluorescens* CIP 106483	Creamery waste	24 h at 25 °C	Pseudomonas Agar Base Oxoid, CM0559
*P. fluorescens* C2 74475	Cheese		
*S. enterica* ser. Derby CIP 60.62T	From LT2 strain	24 h at 37 °C	X.L.D. Medium Oxoid, CM0469
*S. enterica* ser. Derby 2	Carcass		

**Table 2:** Antioxidant activity of raw apple pomace (RP), exhausted pomace (EP), and supercritical CO_2_ extract (APE) determined by ABTS, DPPH and FRAP assays. Results are expressed as mmol TE g^−1^ DW. Values are reported as mean ± standard deviation. Different lowercase letters indicate statistically significant differences as measured by one-way ANOVA followed by Tukey’s post hoc test.

	ABTS	DPPH	FRAP
RP	19.54 ± 0.20 ^b^	43.50 ± 0.77 ^b^	201.14 ± 6.22 ^b^
EP	14.32 ± 0.66 ^c^	12.27 ± 0.41 ^c^	16.49 ± 0.61 ^c^
APE	254.33 ± 1.25 ^a^	123.25 ± 4.32 ^a^	306.15 ± 10.49 ^a^

**Table 3:** Volatile compounds detected and quantified in APE by GC-MS analysis. Results are expressed as µg g^−1^ DW and reported as mean ± standard deviation. The relative abundance of each compound is also reported as a percentage (%) of the total chromatographic area.

Volatile Compound(s)	Quantification (μg g^−1^)	Relative Abundance (%)
Pseudo-ionone	11.90 ± 0.25	0.62
2-Nonenal	16.87 ± 1.66	0.88
Ethyl decanoate	187.14 ± 27.25	9.80
Farnesol (isomer 2)	205.48 ± 36.27	10.76
Octanoic acid	26.49 ± 3.31	1.38
Benzaldehyde	389.50 ± 28.06	20.40
E, Z-2,4-decadienal	51.70 ± 0.97	2.70
1-Octen3-ol	506.89 ± 53.14	26.55
Ethyl benzoate	40.62 ± 4.16	2.12
E, E-2,4-decadienal	38.04 ± 2.72	1.99
2-Octenal	54.98 ± 2.24	2.88
2-Heptenal	62.70 ± 5.31	3.28
Nerylacetone	84.04 ± 8.14	4.40
Decanoic acid	87.04 ± 6.76	4.55
*β*-ionone	17.00 ± 1.41	0.89
Nonanoic acid	18.67 ± 1.07	0.97
2-Phenylethyl acetate	37.43 ± 3.95	1.96
Dodecanoic acid	38.16 ± 3.26	1.99
Ethyl octanoate	34.19 ± 2.88	1.79
TOT	1908.84 ± 192.81	99.91

**Table 4:** Minimum Inhibitory Concentration (MIC) values (mg mL^−1^) of apple pomace extract (APE) against eight bacterial species, including both spoilage bacteria and foodborne pathogens. For each species, MIC was determined against one reference and one wild-type strain. Additionally, the Minimum Biofilm Eradication Concentration (MBEC) and Minimal Biocidal Concentration (MBC) values (mg mL^−1^) of APE are also reported for biofilm-producing strains.

Bacterial Strains	MIC (mg mL^−1^)	MBEC (mg mL^−1^)	MBC (mg mL^−1^)
Gram-positive			
*B. cereus* CIP 66.24T	90	/	/
*B. cereus* 117	Not inhibited	/	/
*E. faecium* CIP 110055	180	45	45
*E. faecium* 1	180	/	/
*L. innocua* ATCC 33090	90	45	45
*L. innocua* 2	90	45	45
*L. monocytogenes* ATCC 13932	90	45	45
*L. monocytogenes* 3	90	45	45
*S. aureus* CIP 65.8T	180	45	45
*S. aureus* 2	90	45	45
Gram-negative			
*E. coli* CIP 105215	90	/	/
*E. coli* 1	45	/	/
*P. fluorescens* CIP 106483	45	/	/
*P. fluorescens* C2 74475	90	/	/
*S. enterica* ser. Derby CIP 60.62T	90	Not inhibited	Not inhibited
*S. enterica* ser. Derby 2	180	/	/

## Data Availability

All the data presented in this study are included in the article. Further inquiries can be directed to the corresponding author.

## Abbreviations

The following abbreviations are used in this manuscript:

ABTS	2,2′-azino-bis (3-ethylbenzothiazoline-6-sulfonic acid)
ANOVA	Analysis of Variance
APE	Apple Pomace Extract
ATCC	American Type Culture Collection
BGC	Biofilm Growth Check
BHI	Brain Heart Infusion
CFU	Colony-Forming Unit
CIP	Collection de l’Institute Pasteur
CLSI	Clinical and Laboratory Standards Institute
DAD	Diode Array Detector
DMAC	4-(dimethylamino)cinnamaldehyde
DMEM	Dulbecco’s Modified Eagle Medium
DMSO	Dimethyl Sulfoxide
DPPH	2,2-diphenyl-1-picrylhydrazyl
DW	Dry Weight
EI	Electron Impact
ELISA	Enzyme-Linked Immunosorbent Assay
EP	Exhausted Pomace
ESI	Electrospray Ionization
FBS	Fetal Bovine Serum
FC	Folin–Ciocalteu
FRAP	Ferric Reducing Antioxidant Power
GAE	Gallic Acid Equivalents
GC-MS	Gas Chromatography–Mass Spectrometry
GM	Growth Medium
HPLC-MS	High-Performance Liquid Chromatography–Mass Spectrometry
LPS	Lipopolysaccharide
MBC	Minimum Biocidal Concentration
MBEC	Minimum Biofilm Eradication Concentration
MHB	Mueller Hinton Broth
MIC	Minimum Inhibitory Concentration
MS/MS	Tandem Mass Spectrometry
MTT	3-(4,5-dimethylthiazol-2-yl)-2,5-diphenyltetrazolium bromide
NIST	National Institute of Standards and Technology
PAC-A2	A2-type proanthocyanidins
PACE	Proanthocyanidins Equivalents
RE	Rutin Equivalents
RP	Raw Pomace
SFE	Supercritical Fluid Extraction
TE	Trolox Equivalents
TFC	Total Flavonoid Content
TNF-α	Tumor Necrosis Factor α
TPAC	Total Proanthocyanidin Content
TPC	Total Phenolic Content
TPTZ	2,4,6-tripyridyl-s-triazine
UV-Vis	Ultraviolet–Visible
